# Pulmonary Rehabilitation Once a Week for One Year in a Patient With Chronic Obstructive Pulmonary Disease

**DOI:** 10.7759/cureus.64049

**Published:** 2024-07-07

**Authors:** Yohei Kubori, Yuji Yasuda, Akira Tamaki

**Affiliations:** 1 Department of Rehabilitation Medicine, Yasuda Clinic, Kyoto, JPN; 2 Department of Respiratory Medicine, Yasuda Clinic, Kyoto, JPN; 3 Department of Physical Therapy, School of Rehabilitation, Hyogo Medical University, Nishinomiya, JPN

**Keywords:** six-minute walking distance, exercise training, rehabilitation, long-term, copd

## Abstract

Pulmonary rehabilitation (PR) has been shown to alleviate dyspnea, increase exercise capacity, and improve quality of life in patients with chronic obstructive pulmonary disease (COPD). However, such PR programs have focused on short-term effects. Thus, this study aimed to report our experience with a COPD patient who underwent PR once a week for one year. An 84-year-old male with stage II COPD, which was classified by the Global Initiative for Obstructive Lung Disease, presented symptoms of dyspnea while walking. The patient underwent PR once a week for one year, which included exercise training, self-management support, instructions on breathing during exertion, and respiratory muscle stretching. Before and after PR, we assessed the patient’s physical function, dyspnea, and quality of life.

For one year, no adverse events were recorded. We observed that the patient’s physical function, dyspnea, and quality of life improved over time. In particular, his six-minute walking distance (6MWD) reached the minimal clinically important difference at three months and the predictive value of 6MWD for healthy adults at six months. The present case showed that a PR program conducted once a week for one year might be feasible and effective.

## Introduction

Pulmonary rehabilitation (PR) has been shown to alleviate dyspnea, increase exercise capacity, and improve quality of life in patients with chronic obstructive pulmonary disease (COPD) [1]. The last Cochrane Library meta-analysis concluded that there was strong evidence supporting the beneficial effects of PR and that no further research was needed in this area [2]. PR programs, which are typically nine to 12 weeks in duration, focus on short-term increases in cardiopulmonary fitness and exercise, and they do not consistently result in sustained increases [3]. Unfortunately, little is known about the long-term effects of PR, especially for elderly people. Thus, the present study aimed to provide and report our experience with PR once a week for one year in a very old patient with COPD.

## Case presentation

**Figure 1:**
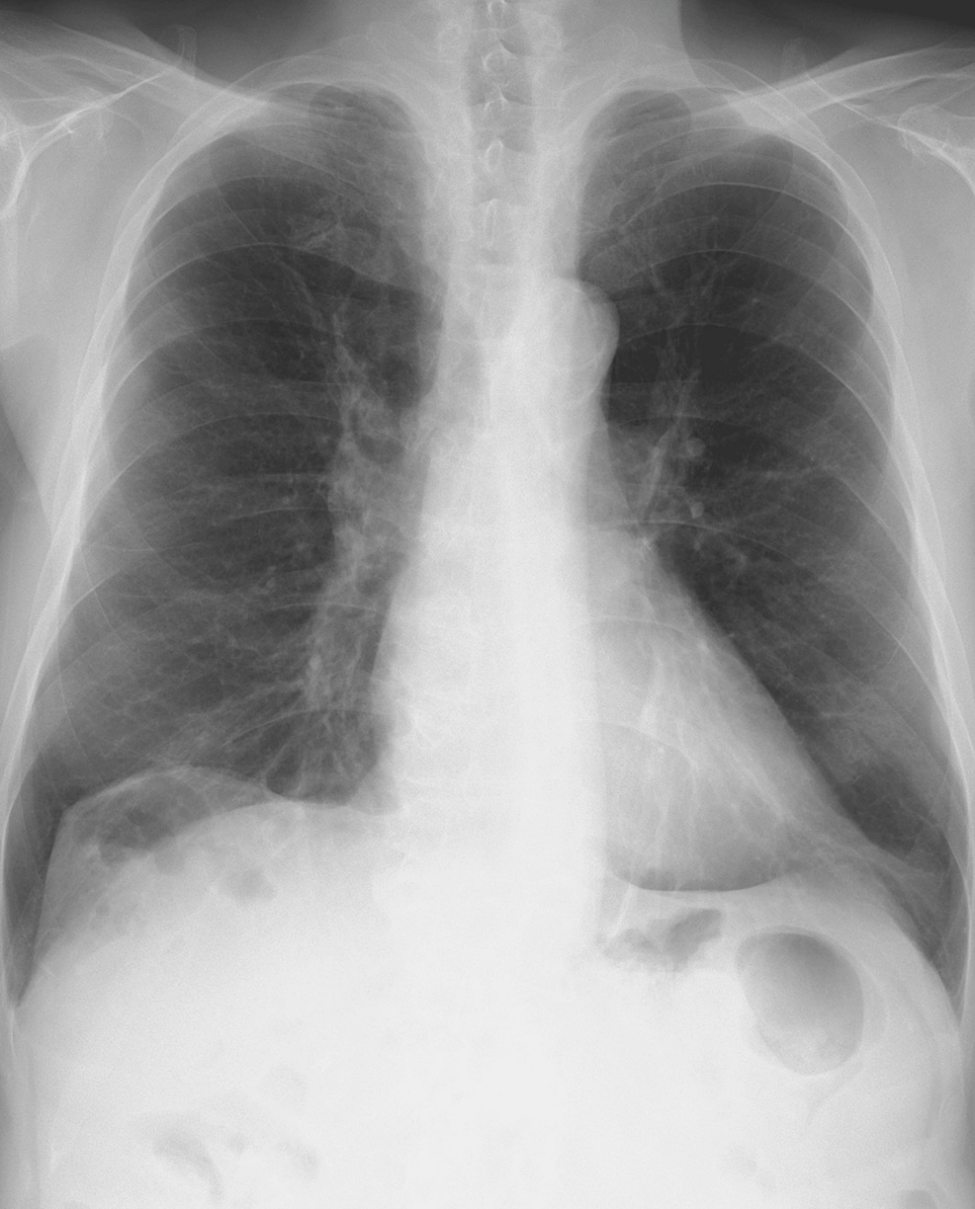
Chest x-ray showed hyperinflation of both lungs.

An 84-year-old male (body mass index: 22.4 kg/m^2^) with COPD presented with symptoms of dyspnea while walking. He was diagnosed with COPD at the age of 78 years and received consultation at the clinic seven years ago because of worsening dyspnea. His smoking history is 60 pack years, and he had two previous exacerbations due to pneumonia. In addition, he had comorbidities including atrial fibrillation, hypertension, and asymptomatic stroke. His condition stabilized after receiving a prescription for single inhaler triple therapy, and PR was initiated to improve his self-management skills and exercise tolerance. The chest x-ray of the patient is shown in Figure 1.

He underwent spirometry before PR, for which the following results were obtained: percent forced vital capacity (FVC)=101.0%; percent forced expiratory volume in 1 s (FEV1.0)=54.0%; and FEV1.0/FVC=51.1%. His airflow limitation was classified as stage II by the Global Initiative for Obstructive Lung Disease. The patient also underwent the symptom-limited incremental cycle ergometry test (ICET) before PR. The peak work rate (PWR), calculated as the maximum work watts maintained for at least 30 s on the ICET, was 54 watts [4]. Before PR and after completion of the 3rd, 6th, and 12th months, we assessed his physical function, dyspnea status, and QoL as follows: hand grip strength, knee extension strength, 4-meter gait speed (4MGS), six-minute walking distance (6MWD), Medical Research Council (MRC) scale score, Barthel Index dyspnea (BId) score, and COPD Assessment Test (CAT) score.

PR was conducted once a week for one year and consisted of exercise training, self-management support, instruction in breathing on exertion, and respiratory muscle stretching. Exercise training included endurance and muscle-strengthening training. The initial exercise intensity of endurance training was 50% of the PWR load achieved on the ICET [4]. The goal for exercise duration in endurance training was set at 20 minutes, and the load was increased to a modified Borg Scale of 4-6 when that goal was achieved. Strength training for limbs was conducted in two sets of 15 repetitions using a normally isotonic leg press machine. Self-management support comprised understanding the use of inhalers, the prevention of exacerbations, daily activity guidelines, movement speed, and the timing of breaks. In particular, in the guidance of daily activities, the daily target number of steps, the content and frequency of indoor exercise were explained in writing in a pamphlet, and the continuity was periodically confirmed. Instruction in breathing consisted of relaxation with breathing control, and pursed-lip breathing during exertion.

**Figure 2:**
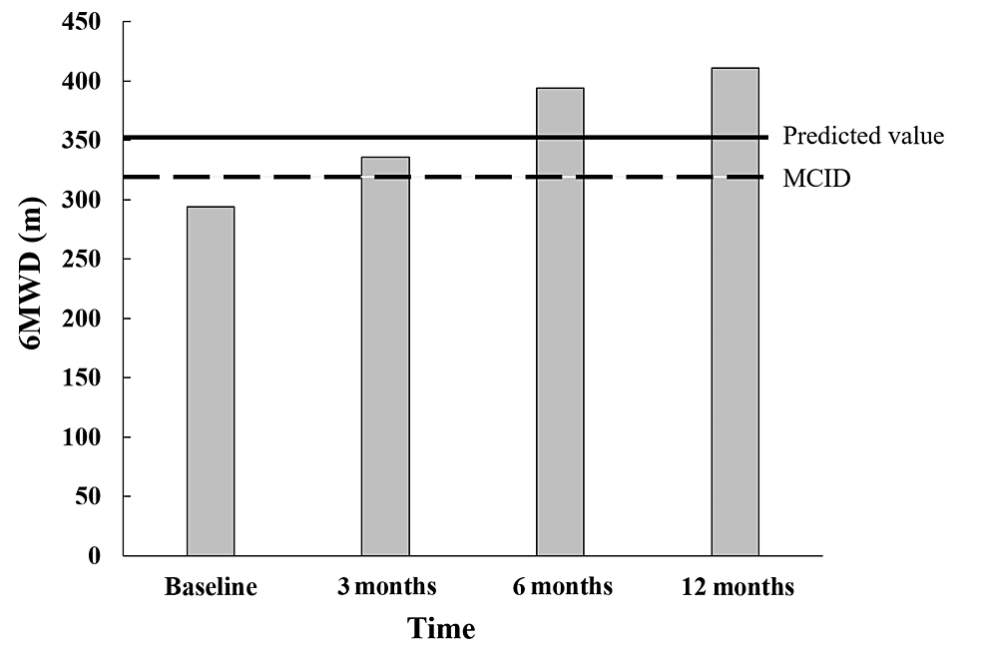
Change in the 6MWD values over time in the PR program. The solid line indicates the predictive value of the 6MWD for healthy adults. The dashed line indicates the minimal clinically important difference. 6MWD: 6-min walk distance; PR: pulmonary rehabilitation

For one year, no adverse events were recorded. The effects of PR on physical function, dyspnea, and QoL are shown in Table 1. We observed that the patient’s knee extension strength, CAT, and 6MWD constantly improved. In particular, the 6MWD reached the minimal clinically important difference (MCID) at three months [5], and the predictive value of the 6MWD for healthy adults at six months (Figure 2) [6]. The 4MGS, MRC scale, and BId also improved over time. The patient provided written informed consent prior to publication of the case details.

**Table 1:** Changes in physical function, dyspnea, and quality of life. 4MGS: 4-meter gait speed; 6MWD: 6-min walk distance; MRC Scale: Medical Research Council Scale; BId: Barthel Index dyspnea; CAT: COPD Assessment Test

Variables	Baseline	Post-pulmonary rehabilitation		
		3 months	6 months	12 months
Physical function				
Hand grip strength (kg)	30.5	29.5	30.5	31.4
Knee extension strength (kg)	35.9	40.45	43.35	52.8
4MGS (m/s)	0.71	0.90	0.97	0.94
6MWD (m)	294	336	394	411
Dyspnea				
MRC scale (point)	2	2	2	1
BId (point)	17	17	8	5
Quality of life				
CAT (point)	15	12	9	8

## Discussion

Recently, there has been growing interest in the role of long-term PR maintenance programs. A long-term PR maintenance program was defined as ongoing supervised exercise at a lower frequency than the initial short-term PR [7]. Previous studies have shown that long-term PR maintenance programs are effective at maintaining short-term PR benefits [3,8,9]. Moreover, Foglio et al. proposed another maintenance strategy consisting of repeated yearly short-term PR programs [10]. These long-term PR maintenance programs have been shown to maintain initial short-term PR benefits; however, significant improvements have not been observed. Thus, few studies have evaluated long-duration programs aimed not only at maintaining but also at improving clinically relevant outcomes.

The present patient demonstrated an improvement in his 6MWD at three months, which exceeded the MCID [11-13]. Similarly, his knee extension strength, 4MGS, and CAT exceeded the MCID at three months. Furthermore, these functions continuously improved for one year. On the other hand, there was no change in the MRC or BId at three months. Supervised exercise training at least twice weekly is usually recommended for COPD patients [14]. Thus, differences in the frequency of PR might affect the change of dyspnea. However, although the PR intervention frequency was only once a week, this patient experienced improvements in dyspnea at 12 months. In particular, the BId reached the MCID at six months [15]. Therefore, even at a low frequency, PR continued consistently for a long-term duration might be effective. Reis et al. investigated the effects of a long-term PR program and suggested that receiving PR three times a week for two years leads to a progressive improvement in QoL and exercise tolerance [16]. On the other hand, it has been reported that a requirement for frequent adherence to PR affects compliance with the program [17-19]. Sahin and Naz reported that transportation problems are the second most common reason for impaired program participation or completion of PR [17]. Therefore, it is preferable to plan a low-frequency PR program as much as possible. The present case showed that conducting a PR program once a week for one year might be feasible and effective.

## Conclusions

The major finding of this study is that receiving PR once a week for one year may lead to progressive improvements in physical function, dyspnea, and QoL in patients with COPD, even among elderly patients. Notably, the patient participated in PR constantly for one year, and no adverse events were recorded. In the future, large-scale multicenter randomized studies are needed to confirm the long-term efficacy, feasibility, and cost-effectiveness of PR.

## References

[1] McCarthy B, Casey D, Devane D, Murphy K, Murphy E, Lacasse Y (2015). Pulmonary rehabilitation for chronic obstructive pulmonary disease. Cochrane Database Syst Rev.

[2] Lacasse Y, Cates CJ, McCarthy B, Welsh EJ (2015). This Cochrane Review is closed: deciding what constitutes enough research and where next for pulmonary rehabilitation in COPD. Cochrane Database Syst Rev.

[3] Ries AL, Kaplan RM, Myers R, Prewitt LM (2003). Maintenance after pulmonary rehabilitation in chronic lung disease: a randomized trial. Am J Respir Crit Care Med.

[4] Kozu R, Senjyu H, Jenkins SC, Mukae H, Sakamoto N, Kohno S (2011). Differences in response to pulmonary rehabilitation in idiopathic pulmonary fibrosis and chronic obstructive pulmonary disease. Respiration.

[5] Holland AE, Hill CJ, Rasekaba T, Lee A, Naughton MT, McDonald CF (2010). Updating the minimal important difference for six-minute walk distance in patients with chronic obstructive pulmonary disease. Arch Phys Med Rehabil.

[6] Troosters T, Gosselink R, Decramer M (1999). Six minute walking distance in healthy elderly subjects. Eur Respir J.

[7] Malaguti C, Dal Corso S, Janjua S, Holland AE (2021). Supervised maintenance programmes following pulmonary rehabilitation compared to usual care for chronic obstructive pulmonary disease. Cochrane Database Syst Rev.

[8] Blervaque L, Préfaut C, Forthin H (2021). Efficacy of a long-term pulmonary rehabilitation maintenance program for COPD patients in a real-life setting: a 5-year cohort study. Respir Res.

[9] Güell MR, Cejudo P, Ortega F (2017). Benefits of long-term pulmonary rehabilitation maintenance program in patients with severe chronic obstructive pulmonary disease. Three-year follow-up. Am J Respir Crit Care Med.

[10] Foglio K, Bianchi L, Bruletti G, Porta R, Vitacca M, Balbi B, Ambrosino N (2007). Seven-year time course of lung function, symptoms, health-related quality of life, and exercise tolerance in COPD patients undergoing pulmonary rehabilitation programs. Respir Med.

[11] Iwakura M, Okura K, Kubota M, Sugawara K, Kawagoshi A, Takahashi H, Shioya T (2021). Estimation of minimal clinically important difference for quadriceps and inspiratory muscle strength in older outpatients with chronic obstructive pulmonary disease: a prospective cohort study. Phys Ther Res.

[12] Kon SS, Canavan JL, Jones SE (2014). Minimum clinically important difference for the COPD Assessment Test: a prospective analysis. Lancet Respir Med.

[13] Kon SS, Canavan JL, Nolan CM (2014). The 4-metre gait speed in COPD: responsiveness and minimal clinically important difference. Eur Respir J.

[14] (2023). 2023 Global Initiative for Chronic Obstructive Lung Disease (GOLD) Report. https://goldcopd.org/2023-gold-report-2/.

[15] Vitacca M, Malovini A, Balbi B (2020). Minimal clinically important difference in Barthel Index dyspnea in patients with COPD. Int J Chron Obstruct Pulmon Dis.

[16] Reis LF, Guimarães FS, Fernandes SJ, Cantanhede LA, Dias CM, Lopes AJ, De Menezes SL (2013). A long-term pulmonary rehabilitation program progressively improves exercise tolerance, quality of life, and cardiovascular risk factors in patients with COPD. Eur Phys Rehabil Med.

[17] Sahin H, Naz I (2018). Why are COPD patients unable to complete the outpatient pulmonary rehabilitation program?. Chron Respir Dis.

[18] Hayton C, Clark A, Olive S (2013). Barriers to pulmonary rehabilitation: characteristics that predict patient attendance and adherence. Respir Med.

[19] Mathar H, Fastholm P, Lange P, Larsen NS (2017). Why do patients decline participation in offered pulmonary rehabilitation? A qualitative study. Clin Rehabil.

